# In Search of Novel Targets for Heart Disease: Myocardin and Myocardin-Related Transcriptional Cofactors

**DOI:** 10.1155/2012/973723

**Published:** 2012-05-17

**Authors:** Alexander T. Mikhailov, Mario Torrado

**Affiliations:** Institute of Health Sciences, University of La Coruña, Campus de Oza, Building El Fortin, Las Jubias Street s/n, La Coruña 15006, Spain

## Abstract

Growing evidence suggests that gene-regulatory networks, which are responsible for directing cardiovascular development, are altered under stress conditions in the adult heart. The cardiac gene regulatory network is controlled by cardioenriched transcription factors and multiple-cell-signaling inputs. Transcriptional coactivators also participate in gene-regulatory circuits as the primary targets of both physiological and pathological signals. Here, we focus on the recently discovered myocardin-(MYOCD) related family of transcriptional cofactors (MRTF-A and MRTF-B) which associate with the serum response transcription factor and activate the expression of a variety of target genes involved in cardiac growth and adaptation to stress via overlapping but distinct mechanisms. We discuss the involvement of MYOCD, MRTF-A, and MRTF-B in the development of cardiac dysfunction and to what extent modulation of the expression of these factors *in vivo* can correlate with cardiac disease outcomes. A close examination of the findings identifies the MYOCD-related transcriptional cofactors as putative therapeutic targets to improve cardiac function in heart failure conditions through distinct context-dependent mechanisms. Nevertheless, we are in support of further research to better understand the precise role of individual MYOCD-related factors in cardiac function and disease, before any therapeutic intervention is to be entertained in preclinical trials.

## 1. Introduction

Heart failure (HF) is the common end-stage condition of various cardiovascular disorders characterized by a progressive decrease in cardiac output combined with insufficient or absent compensatory mechanisms. HF is a major public health problem affecting 15 million patients in the European Union [1]. In descriptive terms, HF can be classified as acute or chronic, congestive, systolic or diastolic, with high or low output, left sided or right sided, and backward or forward. Prevalent causes of sporadic (nonfamilial) HF include myocardial infarction, hypertension, and ischemic or dilated cardiomyopathy. Diuretics, ACE (angiotensin-converting enzyme) inhibitors, and angiotensin-II receptor blockers improve survival in patients with chronic HF but do not prevent cardiomyocyte molecular and structural deterioration (reviewed in [2]). Despite progress in both clinical investigation and basic cardiovascular research (see [3–5]), targeting the molecular mechanisms that promote and sustain HF development in patients still remains elusive (albeit with certain progress, see [6, 7]).

Extensive research over the past 10 years has begun to provide significant advances in our understanding of the interplay between heart development and disease [8–11]. The important step in this direction is illustrated by recent studies of gene regulatory networks (GRNs) operating in cardiac muscle cells [12–15]. The unraveling of GRNs in both normal and diseased heart offers the opportunity to identify single key factors for regulation of the gene expression networks which are ubiquitously altered in failing myocardium, regardless of the etiology of HF [16].

The major players involved in cardiac GRNs are transcription factors, notable among which is the serum response factor (SRF; [15]). SRF, a member of the MADS (*M*CM1, *A*G, *D*EFA, *S*RF) box family of transcription factors, binds as a dimer to specific sites (known as CArG boxes located within serum response elements) on DNA driving the expression of hundreds of target genes in a wide array of cell types and tissues, from brain to cardiac muscle [17–19]. Although ubiquitously expressed, SRF plays specialized roles in different cellular environments. One of the mechanisms by which SRF regulates gene expression in a cell-specific manner is through SRF recruitment of several cofactors which are specifically expressed in a given cell type. In different muscle cell types (cardiac, smooth, skeletal), cell-specific regulation of the SRF-dependent gene expression is achieved by SRF binding of specific coactivators, such as myocardin (MYOCD) and myocardin-related transcription (MRTF) cofactors, MRTFA and MRTFB (reviewed in [20–23]).

The data from both animal models and clinical research in patients provided evidence of pathological consequences of both SRF redundancy and deficiency in the adult heart. In fact, SRF hyperexpression can lead to pathological hypertrophy, while inhibition of SRF activity can result in development of dilated cardiomyopathy (reviewed in [18]). In light of the involvement of SRF in adverse cardiac remodeling, it has been of interest to explore the potential contributions of MYOCD family members, as SRF coactivators, to heart disease. As a result, the data accumulated over the last years clearly implicate the MYOCD protein family in several common forms of adult cardiac disease, such as pathological cardiac hypertrophy, myocardial infarction, and HF [24]. Moreover, the *MYOCD *gene expression is upregulated in circulating blood cells of patients with sporadic hypertrophic cardiomyopathy [25] or hypertension [26].

There have been a series of excellent reviews on molecular and functional characterization, and regulation of MYOCD and MRTFs in different muscle and nonmuscle cell types [20, 24, 27–29], and these essential facets will be commented relatively briefly here. Instead, this paper emphasizes recent insights into the involvement of transcriptional cofactors of the MYOCD family in cardiac dysfunction and to what extent experimental modulation of these factors' expression *in vivo* can correlate with cardiac disease outcome.

## 2. Myocardin Family Proteins: Functional Domains and Transcription Factor Binding Motifs

The myocardin protein family includes (as mentioned above) MYOCD itself and two MYOCD-related transcription factors, MRTF A and B (also referred to as MAL/MKL1 and MAL16/MKL2, resp.). Properties of the MYOCD-related family of SRF-binding cofactors have been characterized most extensively in MYOCD itself, as the founding member, and are presumed to be shared among other MYOCD family members. Although sometimes members of the MYOCD protein family are called “transcription factors” (see, e.g., [24]), they do not bind to DNA itself. Instead, they form a ternary complex with SRF anchored to the CArG box of promoters of cardiac and smooth muscle (SM) contractile genes.

**Figure 1:**
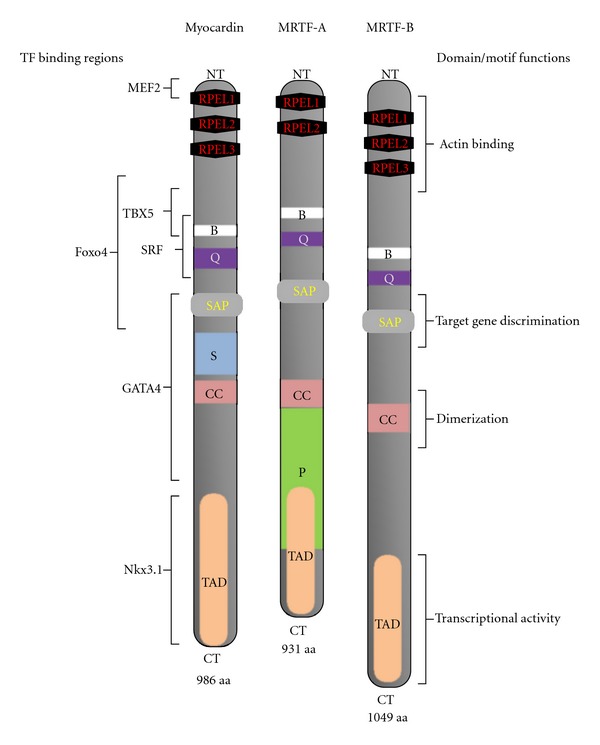
The myocardin family of transcriptional cofactors: protein signatures, domains, and functional sites. The domain/motif structures of the human proteins are shown: RPEL 1-3 (RPEL domain), B: basic domain, Q: glutamine-rich region, SAP: SAP domain; S: serine-rich region; CC: coiled-coil (leucine zipper-like) motif; P: proline-rich region; TAD: transcription activation domain. The regions essential for binding of transcription factors (TF). MEF2: myocyte enhancer factor-2 [32], TBX5: T-box transcription factor 5 [33], FOXO4: forkhead box protein O4 [34], GATA-4: member of GATA family of zinc finger transcription factors [35], NKX-3.1: homeobox NK-3 transcription factor-1 [36]. NT/CT: amino/carboxyl terminus.

MYOCD is a SAP (scaffold-attachment factor A/B, Acinus, PIAS) domain protein that was discovered by Olson's group during an *in silico* search for genes underlying early heart development [30].* Mrtf-A* and *Mrtf-B* were initially isolated by Wang and colleagues [31] on screening cDNA libraries with *Myocd*-related probes. All of them show a similar domain organization (Figure 1) consisting of RPEL motifs, a basic domain, a glutamine-rich region, a SAP domain, a leucine zipper-like region, and a transcription activation domain.

The N-terminal region of MYOCD proteins contains several RPEL motifs (known also as RPEL domain) capable of interacting with globular actin. Differences between RPEL domains appear to define different nucleocytoplasmic transport activities of MYOCD family proteins: MYOCD is constitutively located in the nucleus whereas its family members, MRTF-A and MRTF-B, mostly reside in the cytoplasm and translocate to the nucleus in response to actin polymerization (for more details see [37, 38]). Recently, it has been shown that nuclear accumulation of MYOCD family members also depends on their different relative affinities for nuclear import factors [39, 40]. Of note, the MEF2 (myocyte enhancer factor 2) binding motif located in RPEL1 is unique to MYOCD; transfer of this MEF2 binding motif to MRTF-A confers the ability to co-activate the MEF2 transcription factor [32]. The other SAP domain protein (named MASTR) shares this motif with MYOCD and acts as a MEF2 co-activator [41].

MRTF-A and MRTF-B share strong sequence homology with MYOCD in the basic and Q-rich domains, which are responsible for SRF binding. The SAP domain, characteristically observed in diverse nuclear proteins involved in chromatin organization/remodeling, is not required for interaction of MYOCD proteins with SRF. However, mutations in the SAP domain of MYOCD affect activation of some SRF-dependent gene targets, but not others, suggesting a role of this domain in target gene discrimination. Although all MYOCD proteins have a highly conserved transactivation domain at their C-terminal region, MRTF-A activates CArG-dependent SM-gene promoters reaching levels similar to those induced by MYOCD itself, whereas MRTF-B is less effective in this regard. Several consensus regions of MYOCD have been shown to be putative sites for binding of transcription factors, other than SRF, which are also involved in regulation of cardiac gene transcription and expression or associated with the response of cardiac tissue to stress (see Figure 1, MEF-2, TBX5, GATA4, and NKX3.1). Forthcoming studies should reveal the full potential of the MYOCD family in cardiac transcription regulatory networks.

Formally viewed, a high degree of domain similarity between members of the MYOCD family indicated they could have similar or overlapping functions in developing and adult heart. However, evidence of similar domain organization alone does not prove that MYOCD family members do in fact perform identical or similar functions or actions in the heart. Basic assumptions regarding similarities and differences in their expression and functions in cardiac tissue are discussed next.

## 3. Myocardin Family Proteins and Heart Development: Lessons from Mouse Knockout Models

Complete loss-of-function (total/global or constitutive gene knockout) experiments suggest the function(s) of a gene of interest through the phenotype that results from its deficiency in mouse mutants and, by extrapolation, in humans. Even an early embryonic lethal phenotype might be informative enough to predict a gene as essential for a given fetal tissue/organ, but roles the gene may play later in development as well as in postnatal/adult life remain unknown. To overcome these problems, techniques have been developed to create conditional knockout models, in which a gene of interest can be inactivated in a spatially and temporally controlled fashion. Several such approaches have been used to evaluate to which extent the members of the MYOCD family of transcriptional cofactors are involved in molecular and cellular processes underlying heart development and maturation.

The members of the MYOCD family are coexpressed in cardiac as well as different SM lineages during early mouse embryonic development [30, 31]. Although a single gene-null mutation in *Myocd* and *Mrtf-B *resulted in a wide range of tissue abnormalities and embryonic lethality, here we will consider only structural and functional consequences of inactivation of these genes in the heart and associated structures. (For accounts of the wider spectrum of perturbations observed in these knockouts, see references provided in Table 1). 

In mice, the complete loss of *Myocd* leads to a severe early developmental, lethal phenotype from a failure in vascular SM differentiation [42]. However, early cardiogenesis was unaffected in the *Myocd*-null mice. Heart development in *Myocd* knockouts could be partially rescued by redundant activities of *Mrtf-A* and/or *Mrtf-B*. Although repeatedly stated, this suggestion has not yet been tested experimentally. By contrast, despite the fact that both *Myocd* and *Mrtf-B* are coexpressed in embryonic vascular SM cells, *Myocd*-null mice lack differentiated SM cells in the dorsal aorta and placental vasculature.

Due to the early lethal phenotype of the *Myocd*-null embryos, a conditional knockout approach was taken for selective ablation of *Myocd* in developing heart. Cardio-restricted inactivation of the *Myocd* gene did not alter heart development. However, after birth mutant mice with a conditionally inactivated *Myocd* gene develop dilated cardiomyopathy (DCM) accompanied by impaired cardiomyocyte structural organization and severely depressed systolic function [43]. In chimeric *Myocd* knockout mice, generated by injection of *Myocd*-null embryonic stem cells into wide-type blastocysts, *Myocd*-null cells fail to contribute to formation of ventricular myocardium, although these cells were phenotypically normal and did not display ultrastructural alterations [50]. Moreover, these results imply that *Myocd* is absolutely required for functional development of ventricular myocardium and can play a pivotal role in the response of the heart to stressful stimuli after birth.

In contrast to *Myocd*-null embryos, *Mrtf-B* homozygous null or hypomorphic knockout embryos express cardiovascular phenotypic abnormalities resembling those observed in human patients with congenital heart disease. Although knockout models used in these studies resulted in phenotype of differing severity, thin-walled ventricular myocardium and ventricular septal defects were observed in each model setting. Abnormal patterning of the branchial arch arteries [47] and *truncus arteriosus* [48, 49] are also among the other common manifestations (see Table 1). Although the consequences of depleting *Mrtf-B* specifically within the myocardium have not yet been reported, the results of global *Mrtf-B* knockouts demonstrate that this factor is absolutely required for embryonic heart development, specifically within ventricular and cardiac outflow tract compartments.

The role of *Mrtf-A* in cardiovascular development is still unclear. Two groups have independently shown that *Mrtf-A* knockouts are viable and can reach adulthood without obvious cardiac or other organ abnormalities; only knockout females possess mammary gland dysfunction due to the failure of mammary myoepithelial cells to differentiate [44, 46]. Although *Mrtf-A* is not absolutely required for heart development, it may play a particular role in the survival of myocardial cells during certain periods of development: a set of *Mrtf-A* null mouse embryos suffered dilatation of all cardiac chambers and died at midgestation stages [46]. In addition, young adult mice lacking *Mrtf-A* show a significantly weaker hypertrophic response to both acute and chronic pressure overload suggesting *Mrtf-A* is involved in hypertrophy signaling pathways in the postnatal heart [45, 51].

Mice lacking each member of the *Myocd* family exhibit distinct global phenotypes during development [24, 28]. Within the cardiovascular system, knockout mutations in the *Myocd*, *Mrtf-A,* or *Mrtf-B* gene specifically affect distinct aspects of heart formation at different stages of development. Inactivation of *Mrtf-B* drastically alters pattern formation in the developing heart that can lead to congenital heart malformations, whereas deficiency in *Myocd* and *Mrtf-A* becomes phenotypically evident in rather mature heart resulting, respectively, in development of severe DCM or poor hypertrophic response. Whether these factors are also involved in adult heart function and, if so, how these factors modulate the responsiveness of the heart to stress signaling is highlighted below.

## 4. Myocardin Family Proteins in Adult Heart

Once heart development is completed, postnatal functional maturation of the heart takes over extending the use of cardiac GRNs to adulthood raising the possibility that some forms of adult heart disease can result from adult-related alterations in components of these regulatory pathways [52–55].

**Figure 2:**
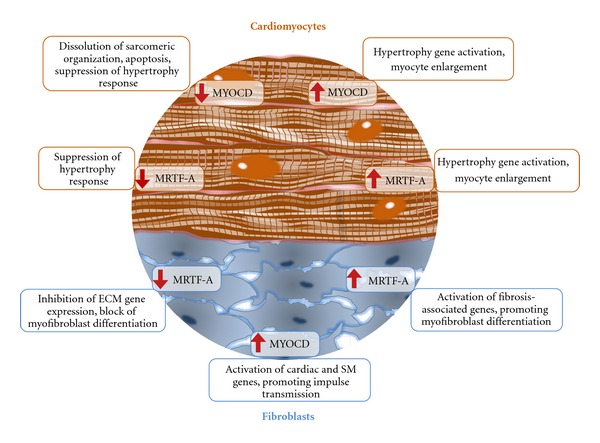
Dissecting the cell-autonomous roles of myocardin and MRTF-A in adult ventricular myocardium. Red arrows indicate up- or downregulation. In cardiomyocytes, forced expression of either *Myocd* or *Mrtf-A* induces hypertrophic gene expression and myocyte enlargement, whereas inhibition of any of these factors markedly attenuates hypertrophic responses. Although both factors display prohypertrophic activities, MYOCD, but not MRTF-A, is absolutely required for myocyte structural integrity and survival [43]. In cardiac fibroblasts, forced expression of *Mrtf-A* activates profibrosis gene expression and myofibroblast differentiation, whereas a loss of functional *Mrtf-A* leads to opposite effects. Forced *Myocd* expression stimulates both SM (including markers of myofibroblast differentiation) and cardiac genes (including cardiac ion channels and connexins) in ventricular fibroblasts.

Members of *MYOCD* gene family are expressed in normal and diseased adult myocardium. The ventricular myocardium, a highly ordered tissue structure, consists of different cell types (cardiac myocytes, fibroblasts, and vascular endothelial and SM cells) which all are involved in myocardial remodeling in response to physiological and pathological stresses. Here, we will discuss arguments which suggest the possible functions of MYOCD family factors in both cardiomyocyte and fibroblast cell compartments of adult myocardium (Figure 2).

### 4.1. MYOCD

MYOCD is absolutely required for maintenance of adult heart function. Cardio-restricted inactivation of the *Myocd* gene in the adult mouse heart resulted in development of severe four-chambered DCM as a result of massive myocyte loss via apoptosis and replacement fibrosis. *Mrtf-B*, showing nonaltered cardiac expression, did not rescue the DCM phenotype in *Myocd*-knockdown mice. It was found in this model system that *Myocd* itself functions in a cardiomyocyte autonomous manner to regulate myocyte gene expression and myofibrillar organization and to prevent cardiomyocyte apoptosis in response to physiological hemodynamic stress [43].

In light of these results, it is not surprising that MYOCD has previously been implicated in remodeling of ventricular myocardium in response to either physiological or pathological pressure-overload hypertrophy [56–58]. In addition, MYOCD is necessary for and mediates the hypertrophic response of ventricular myocardium to a variety of stimuli such as phenylephrine, endothelin, isoproterenol, aldosterone, angiotensin II, insulin and insulin-like growth factors, and beta-transforming growth factor (TGF-*β*). Forced expression of *Myocd* in cultured cardiomyocytes increases cell size and expression of molecular markers of cardiac hypertrophy, whereas knockdown of *Myocd* attenuates hypertrophic response capacity of cardiomyocytes [58–63]. Hypoxia in neonatal cardiomyocytes increases levels of MYOCD and ROS (reactive oxygen species) resulting in cellular hypertrophy, which can be partly reversed by atorvastatin treatment [64]. Collectively, the experimental data indicate at least two important roles for MYOCD in adult cardiomyocytes: its role in maintenance of structural integrity and survival of cardiomyocytes and its role as a prohypertrophic factor in hypertrophic remodeling induced by genetic models, aortic constriction, and neurohumoral factors.

Despite the popular assumption that *MYOCD* is expressed almost exclusively in cardiac and SM cells, *MYOCD* expression was also detected in human fibroblasts [65] where it is involved in functional differentiation and has a negative role in cell proliferation [66]. In fact, in model cell-based assays overexpression of *Myocd* resulted in inhibition of cell-cycle progression at the G2/M phase and formation of polyploidy cells [67]. MRTF-A and MRTF-B also exert antiproliferative effects on fibroblasts [68].

Expression of *Myocd* in mouse cardiac fibroblasts has not been detected [69] but rat cardiac fibroblasts cultured in a medium conditioned by mesenchymal stem cells (MSCs) did express *Myocd* [70]. In rat cardiac fibroblasts, *Myocd* expression appears to be age dependent and influenced by hypoxia-inducible factor 1*α* [71].

It has been demonstrated that MYOCD-mediated transactivation of target genes is promoter and cell context dependent [72, 73]. MYOCD alone was not sufficient to activate cardiac-specific genes in pluripotent 10T1/2 fibroblasts [74, 75]. Ectopic *Myocd* expression in skeletal muscle-like BC3H1 cell line induced cardiac and SM genes and stimulated formation of SM-like filaments with no evidence for cardiac sarcomerogenesis [76]. Infection of human epicardial cells with an adenovirus vector encoding *Myocd* co-activated both SM and cardiac marker genes but did not lead to the assembly of SM-like filaments [77]. Similarly, forced *Myocd* expression resulted in the activation of a broad range of cardiomyocyte and SM genes in both human foreskin-derived [65] and myocardial scar (MSFs) fibroblasts [78]. Although such *MYOCD*-forced MSFs did not acquire the cardiomyocyte structural and functional phenotype, they became capable of conducting an electrical impulse. *In vitro*, placing of *Myocd*-forced fibroblasts between two separate cardiomyocyte fields resulted in resynchronization of two dyssynchronously beating cardiomyocyte areas [78]. Of note, forced expression of *Myocd* enhances the therapeutic effect of MSCs implanted into the infracted area of postmyocardial infarction (MI) in mice [79].

*Myocd* expression is upregulated by TGF-*β*1 in both fibroblasts [66] and SM cells [80], and MYOCD participates in TGF-*β*1 signal-transducing pathways to activate SM gene transcription [81, 82]. Cardiac TGF-*β*1 expression is upregulated in both animal models of MI and ventricular hypertrophy and patients with hypertrophic or DCM [83]. Although unproven as yet, it is tempting to speculate that in the infarcted heart TGF-*β*1 induction of *Myocd* expression might contribute to fibroblast-to-myofibroblast transition. In this sense, a very recent report provides direct evidence that both MRTF-A and MRTF-B are key regulators of TGF-*β*1-induced fibroblast-to-myofibroblast transition [84].

### 4.2. MRTF-A

Consistent with its discovery as a MYOCD-related factor, it has initially been suggested that MRTF-A plays a potentially important role in the heart [31]. However, evidence of this has been found only recently with the use of cardiac dysfunction models in adult *Mrtf-A* null mice. It is appropriate to keep in mind that under basal conditions *Mrtf-A* null mice did not manifest any obvious cardiac structural or physiological deficiencies [44], and inactivation of *Mrtf-A* expression was not associated with changes in *Myocd* or *Mrtf-B* expression in mutant hearts [45].

*In vivo* studies, using the aortic banding model, indicated that loss of *Mrtf-A* inhibits or diminishes activation of the hypertrophic gene program induced in left ventricular (LV) myocardium by mechanical pressure overload. At the morphological level, a degree of LV hypertrophy was also decreased in *Mrtf-A* mutant mice with experimentally induced chronic pressure overload as compared to controls. The same effects were also observed in *Mrtf-A*-null mice subjected to chronic angiotensin II treatment [45]. Taken together, the results identified MRTF-A as a factor mediating prohypertrophic signaling evoked by both mechanical stress and neurohumoral stimulation in ventricular myocardium.

However, it remained uncertain if MRTF-A itself functions in a cardiomyocyte autonomous manner to regulate ventricular hypertrophy. Recent experiments using cultured neonatal cardiomyocytes have demonstrated that overexpression of *Mrtf-A* does induce hypertrophic growth and expression of cardiac hypertrophy marker genes in these cells. In addition, it was found that the expression of both *Mrtf-A* and *Myocd* is induced by hypertrophic signals, whereas dominant-negative mutants of these factors (lacking TAD domain, see Figure 1) attenuate agonist-induced cardiomyocyte hypertrophy. Also, inhibition of endogenous *Mrtf-A* expression reduced phenylephrine, angiotensin-II, and TGF-*β*-induced hypertrophy in neonatal cardiomyocytes [63]. These data, together with those of *Mrtf-A* null mice [45, 51], indicate that besides MYOCD, MRTF-A can play an important role in cardiac hypertrophy as a myocardial prohypertrophic factor.

In the adult heart, *Mrtf-A* is robustly expressed in normal cardiac fibroblasts which are activated to become SM-like myofibroblasts in response to MI. Myofibroblasts strongly express *α*-SM actin and extracellular matrix (ECM) proteins. In the recent report [69], MIs were generated using *Mrtf-A* null and wide-type adult mice by surgical ligation of the left anterior descending coronary artery. Both, a significantly reduced infarct size and attenuated upregulation of the ECM markers (collagens, elastin) in the border zone, were observed in MI *Mrtf-A* null compared to MI wide-type mice. The fibroblast-to-myofibroblast transition is believed to be primarily responsible for fibrotic remodeling of the post-MI heart. The expression of SM markers of myofibroblast activation, as well as a density of myofibroblasts in the MI border zone, was attenuated in *Mrtf-A*-null mice.

Also, *Mrtf-A* null mice display reduced myocardial fibrosis in response to angiotensin treatment. Forced expression of *Mrtf-A* in isolated cardiac fibroblasts was sufficient to activate myofibroblast-associated SM genes that were downregulated in MI *Mrtf-A* null hearts [69]. These results suggest that MRTF-A can play an essential role in promoting fibroblast-to-myofibroblast transition and fibrotic remodeling in the post-MI heart. (For more detailed comments and nuances related to MRTF-A-regulated pathways of myofibroblast activation, see [85]).

## 5. Myocardin Family Proteins and Heart Disease

As mentioned above, many lines of evidence have implicated MYOCD as an important regulator of hypertrophic growth of the heart. However, it was not until recently that the ability of MYOCD to promote cardiac hypertrophy *in vivo* was directly examined. In neonatal piglets, *in vivo* forced expression of *MYOCD* in ventricular myocardium resulted in activation of a set of fetal cardiac and SM genes associated with impaired systolic performance although no significant change in heart-to-body weight ratio was detected in *MYOCD*-transfected compared with control-sham animals [86]. In neonatal piglets, the right (RV) and left (LV) ventricles display such a different degree of hypertrophy that the RV can be used as a slower heart-matched reference-control for the much more rapidly thickening LV [87, 88]. Nevertheless, expression of *MYOCD* was found to be similar in both the LV and RV of porcine as well as human neonates [56]. The simplest interpretation of these data is that although MYOCD does upregulate the expression of a set of SRF/MYOCD-dependent genes in response to hypertrophy signals, it alone is not sufficient to discriminate among different patterns of LV/RV remodeling in the adult heart *in vivo*.

In addition to its role in adaptive gene expression and maintenance of cardiac function, MYOCD has also been implicated in the response of the adult heart to pathological stresses during severe hypertrophic remodeling [58, 89] and at end-stage HF due to dilated, ischemic, and cardiotoxic CM [56, 58, 90]. All of these conditions are characterized by upregulation of *MYOCD* expression in failing LV myocardium. In addition, MYOCD SM targets are also upregulated in failing myocardium in both animal models and patients [91]. Although this correlative evidence links MYOCD signaling to the acquisition of pathological conditions, the role that *MYOCD* gene activation plays in HF conditions *in vivo* has remained unclear. Recently, we attempted to integrate upregulated *MYOCD* signaling into the pathogenesis of HF, using targeted RNAi-mediated *MYOCD* gene inhibition in the porcine model of diastolic HF (DHF). *In vivo* silencing of endogenous *MYOCD* at mid-advanced stages of DHF resulted in downregulation of *MYOCD*-dependent SM-gene expression in failing myocardium [92]. Such adjusting of *MYOCD* and SM-target expression levels to the range of physiological variation resulted in restoring diastolic function and extending the survival of failing animals without compromising the physiological functions of MYOCD signaling [43] as a part of the adaptive response of the heart to stress.

To the best of our knowledge, the role of MRTF-A and MRTF-B in failing myocardium was not evaluated in either study. Nevertheless, the data from experiments with *Mrtf-A* null mice strongly suggest that modulation of *Mrtf-A* expression in hypertrophy-induced [45] or post-MI [69] failing myocardium could be promising from therapeutic point of view. Of note, mRNA splicing of the *MRTF-B* gene was altered in myocardial samples from patients with ischemic cardiomyopathy undergoing heart transplantation [93].

## 6. Conclusion and Expectations

Currently, a majority of the gene therapy assays are conducted using a single-candidate gene approach. There are two main strategies applied to the search for target genes for the treatment of HF. One is identification of terminal effector genes involved in the advanced and end-stage HF in patients. Another is to establish a new target for HF, searching for members of gene regulatory circuits, each of which can play a role in the control of a branch of terminal effector genes in the network, in both normal and diseased heart. Given the plethora of genes regulated by partnership of SRF and members of the MYOCD family in muscle cells, this balanced regulatory network plays a central role in normal heart development and adult heart function, whereas dysregulation of SRF/MYOCD/MRTF-dependent gene expression contributes to numerous disease models of the cardiovascular system.

Experimental manipulation of expression of MYOCD family genes has allowed the development of new animal models to study the mechanisms of DCM (MYOCD), cardiac hypertrophy, post-MI fibrosis (MRTF-A), and congenital heart disease (MRTF-B). Knowledge gained from these studies will guide the development of novel therapies for the treatment and prevention of HF development. Mice with a cardiomyocyte-specific deficiency in *Myocd* could aid in the development of apoptosis-blocking therapies for HF. *Mrtf-A* null models indicate that *Mrtf-A* inhibition might be therapeutically beneficial in post-MI cardiomyopathy settings. A normalization of activated MYOCD signaling in ventricular myocardium at midstages of HF development can improve impaired ventricular function.

Several cardiovascular-enriched microRNAs (miRNAs), both downstream and upstream to *Myocd*, were found to be involved in the SRF/MYOCD regulatory network [94–98]. In this sense, we suggest a promising therapeutic role for miRNA mimics/inhibitors to modify exaggerated MYOCD signaling in HF settings. In fact, recent data revealed that miR-9 can suppress *Myocd* translational activity *in vitro* and administration of the miR-9 mimic can attenuate cardiac hypertrophy remodeling *in vivo* [97].

Finally, an understanding of synergistic or additive interactions between MYOCD family factors in principal cell types of myocardium (myocytes and fibroblasts) is crucial before any therapeutic intervention could be entertained in preclinical trials.

## Figures and Tables

**Table 1:** Mouse loss-of-function studies of *Myocd*-related family.

Gene	knockout	Embryonic cardiovascular phenotype	Embryonic phenotype	After-birth phenotype	Reference
*Myocd*	Constitutive	Block in SMC differentiation; heart development is obviously normal	Lethal; early-to-mid-gestation	No survivors	[42]

*Myocd*	Conditional, heart restricted	Obviously normal heart development	Obviously normal	DCM, HF	[43]

*Mrtf-A*	Constitutive	No obvious abnormalities	Obviously normal	Myoepithelial defects of the mammary gland;poor hypertrophic response	[44]
					[45]

*Mrtf-A*	Constitutive	65%-no obvious abnormalities;	65%-obviously normal	Myoepithelial defects of the mammary gland	
		35%-dilated heart	35%-lethal, early-to-mid-gestation		[46]

*Mrtf-B*	Constitutive	Double outlet RV, ventricular septal defects, thin-walled myocardium, abnormal patterning of the branchial arch arteries	Lethal; midgestation	No survivors	[47]

*Mrtf-B*	Constitutive	Double outlet RV, cardiac outflow tract defects, ventricular septal defects, persistent truncus arteriosus	Lethal, late gestation	No survivors	[48]

*Mrtf-B*	Constitutive	Ventricular septal defects, thin myocardium, truncus arteriosus	Lethal; late gestation	Only a few grossly normal survivors	[49]

RV: right ventricle; HF: heart failure; DCM: dilated cardiomyopathy.
